# Crystal structure of bis­(acetato-κ^2^ *O*,*O*′)di­aqua­[1-(pyridin-2-yl­methyl­idene-κ*N*)-2-(pyridin-2-yl-κ*N*)hydrazine-κ*N* ^1^]terbium(III) nitrate monohydrate

**DOI:** 10.1107/S2056989017009653

**Published:** 2017-07-07

**Authors:** NDiaye Mbossé Gueye, Dieng Moussa, Elhadj Ibrahima Thiam, Aliou Hamady Barry, Mohamed Gaye, Pascal Retailleau

**Affiliations:** a Département de Chimie, Faculté des Sciences et Techniques, Université Cheikh Anta Diop, Dakar, Senegal; b Département de Chimie, Faculté des Sciences, Université de Nouakchott, Nouakchott, Mauritania; c Centre de Recherche e Gif, Institut de Chimie des Substances Naturelles, CNRS–UPR2301, 1 Avenue la Terasse, 91198 Gif sur Yvette, France

**Keywords:** crystal structure, terbium complex, complex, Schiff base, hydrazide, hydrogen bonds

## Abstract

In the title compound, [Tb(C_2_H_3_O_2_)_2_(C_11_H_10_N_4_)(H_2_O)_2_]NO_3_·H_2_O, the Tb^3+^ ion is nine-coordinated in a distorted tricapped trigonal-prismatic geometry by the three N atoms of the tridentate 1-(pyridin-2-yl­methyl­idene)-2-(pyridin-2-yl)hydrazine ligand, four carboxyl­ate O atoms of two chelating acetate groups and two O atoms of the coordinating water mol­ecules. The organic hydrazine ligand is disordered over two orientations with a refined occupancy ratio of 0.52 (3):0.48 (3). All bond lengths in the coordination environment of the Tb^3+^ ion are slightly larger than those observed in the isostructural Y^3+^ and Er^3+^ complexes. In the crystal, the complex cations are linked by pairs of O—H⋯O hydrogen bonds into dimers. These dimers, nitrate anions and non-coordinating water mol­ecules are joined by O—H⋯O and N—H⋯O hydrogen bonds into a three-dimensional structure.

## Abstract 2

The Tb^3+^ ion is nine-coordinated in a distorted tricapped trigonal-prismatic geometry by the three N atoms of the tridentate 1-(pyridin-2-yl­methyl­idene)-2-(pyridin-2-yl)hydrazine ligand, four carboxyl­ate O atoms of two chelating acetate groups and two O atoms of the coordinated water mol­ecules. In the crystal, the complex cations are linked by pairs of O—H⋯O hydrogen bonds into dimers. These dimers, nitrate anions and non-coordinating water mol­ecules are joined by O—H⋯O and N—H⋯O hydrogen bonds into a three-dimensional structure.

## Chemical context   

As a result of their various architectures and numerous applications (Binnemans, 2005 ▸), lanthanide complexes have attracted significant attention, and the synthesis of new complexes of this type has became relevant. Both mononuclear and polynuclear lanthanide complexes reveal specific properties as mol­ecular magnets (Cristóvão & Hnatejko, 2015 ▸), luminescence materials (Lahoud *et al.*, 2016 ▸) and preparates for medical biology (Zhang *et al.*, 2014 ▸). Used as ligands, Schiff bases together with carboxyl­ate anions display large versatility in forming coordination compounds with metal ions and can generate a wide variety of coordination types. Considerable inter­est is afforded to the development of polydentate ligands containing different (hard and soft) N, O or S binding sites, designed to yield special topological structures (Binnemans, 2005 ▸). By appropriate design, the mol­ecular structure of the ligand can be modified in order to coordinate metal ions in diverse modes resulting in specific architectures. The coordination mode also depends on the adopted synthetic procedures. In this context, for synthesis of the terbium(III) complex, the Schiff base 1-(pyridin-2-yl­methyl­idene)-2-(pyridin-2-yl)hydrazine (HL), which provides three soft-donating N atoms from two pyridine rings and the imino function, was used together with acetate anions, which provide hard-donating O atoms, as co-ligands (Neves *et al.*, 1992 ▸; Schwingel *et al.*, 1996 ▸; Gregório *et al.*, 2015 ▸). The ligand HL and acetate groups were used in our previous attempts to prepare new mono- and binuclear lanthanide(III) complexes (Ndiaye-Gueye, Dieng, Thiam, Sow *et al.*, 2017 ▸; Ndiaye-Gueye, Dieng, Thiam, Lo *et al.*, 2017 ▸; Ndiaye-Gueye, Dieng, Lo *et al.*, 2017 ▸). In the present study, mixing of the HL ligand, sodium acetate and hexa­hydrated terbium nitrate yields a nine-coordinated mononuclear complex of Tb^3+^.
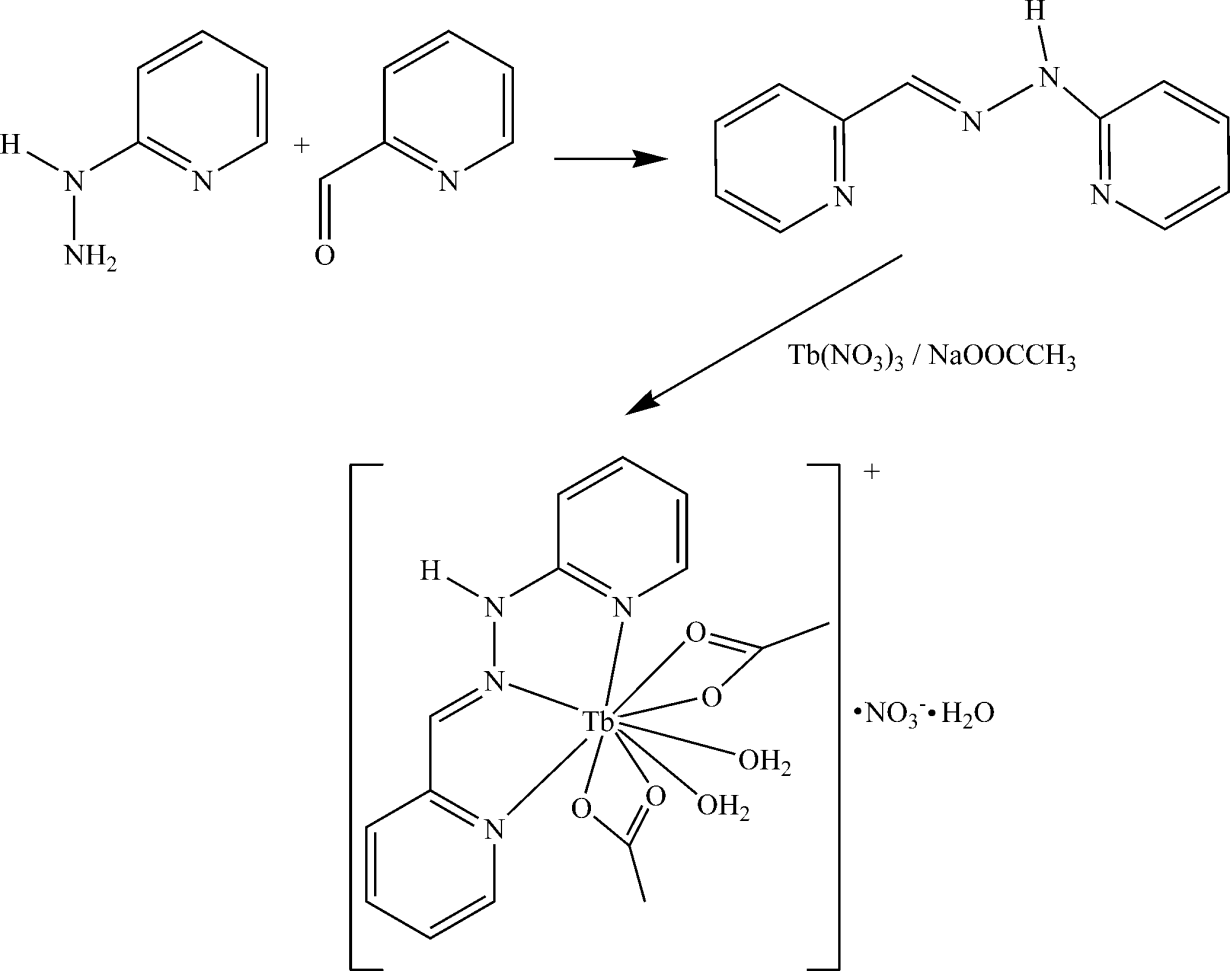


## Structural commentary   

**Figure 1:**
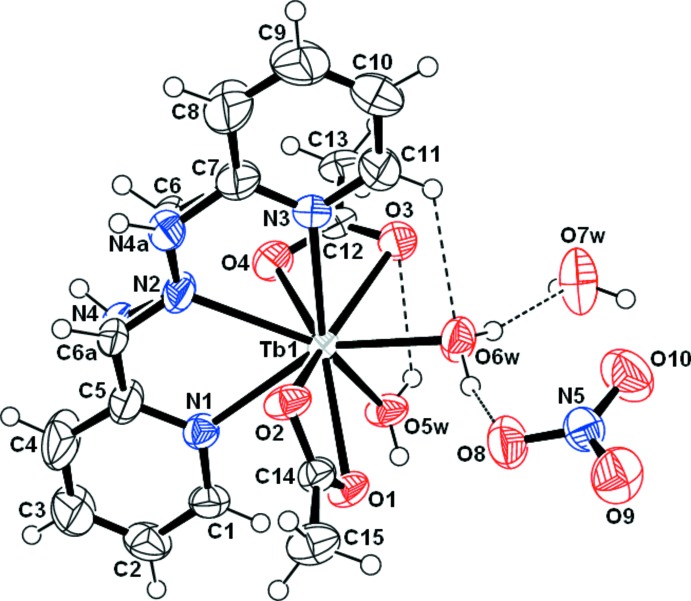
An *ORTEP* view of the title compound, showing some of hydrogen bonds as dashed lines. Displacement ellipsoids are plotted at the 50% probability level.

The crystallographic study shows a 1:1:2 ratio of HL/Tb/acetate in the resulting cationic complex when these components were mixed at room temperature in ethanol with a 1:1:3 ratio. The asymmetric unit comprises a Tb^3+^ ion coordinated by one tridentate HL ligand, two chelating acetate ions, two coordinating water mol­ecules, one non-coordinating nitrate anion and one non-coordinating water mol­ecule (Fig. 1 ▸). The Schiff base acts as a tridentate ligand with three donating N atoms, forming two five-membered chelate rings (TbNCCN and TbNNCN). The Tb^3+^ ion is nine-coordinated and its environment can be described as a distorted tricapped trigonal prism with slanted base faces N1, N2, O2 and O3, O5*W*, O6*W*. The Tb—O(Ac) bond lengths lie within the range 2.401 (3)–2.476 (3) Å (Table 1 ▸) and are comparable to the average value of 2.46 (6) Å for analogous structures from the Cambridge Structural Database (CSD Version 5.38, November 2016; Groom *et al.*, 2016 ▸). The Tb—O*W* bond lengths involving O atoms of the coordinating water mol­ecules of 2.357 (3) and 2.362 (3) Å are also well comparable with the known values [average 2.41 (5) Å from CSD]. In the title structure, the bonds Tb—N differ in length: the distance involving the imino N atom is shorter than those involving the pyridine N atoms: 2.542 (4) Å *vs* 2.574 (4) and 2.588 (4) Å (Table 1 ▸). The same relations between the Tb—N(imine) and Tb—N(Py) bond lengths were observed in the structures of {*N*,*N*′-cyclo­hexane-1,2-diylbis[1-(pyridin-2-yl)methanimine]}-tris­(nitrato)terbium (Chen *et al.*, 2013 ▸) and {(2,9-di­formyl­phenanthroline)bis­[(2-pyrid­yl)hydrazone]}bis­(nitrato)terbium nitrate (Carcelli *et al.*, 2005 ▸), though the absolute values of Tb—N distances of the same kind in these three structures are different. The distances Tb—O(Ac), Tb—O*W* and Tb—N in the title structure are slightly larger (by 0.03–0.04 Å) than the corresponding distances observed in isostructural Y^3+^ and Er^3+^ complexes we recently reported (Ndiaye-Gueye, Dieng, Lo *et al.*, 2017 ▸). These observations can be correlated with the decrease in the unit-cell volume: 1060.5 (2) Å^3^ for Tb^3+^ *vs* 1051.3 (2) Å^3^ for Y^3+^ and 1049.6 (2) Å^3^ for Er^3+^. The bond lengths in the disordered chain C—CH=N—NH—C bridging two pyridine rings are 1.484 (14) and 1.513 (17) Å for C—C, 1.293 (17) and 1.319 (13) Å for C=N, 1.393 (13) and 1.396 (13) Å for N—N and 1.411 (13) and 1.417 (12) Å for N—C. These bonds are slightly longer than observed for this ligand in other complexes. This may be related to the disorder detected for this chain. The dihedral angle formed by the planes of two terminal pyridine rings is 11.0 (4)°.

## Supra­molecular features   

**Figure 2:**
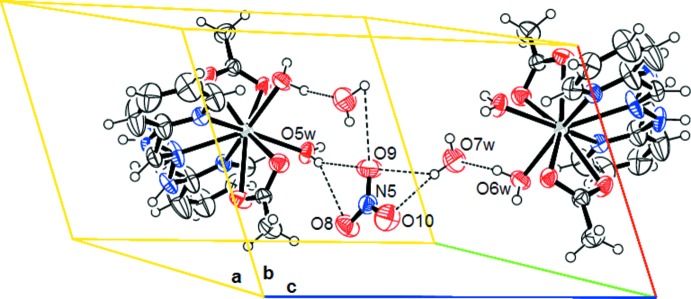
The packing showing the hydrogen bonds as dashed lines.

The crystal structure is stabilized by hydrogen bonds giving rise to a three-dimensional network (Table 2 ▸). The complex cations are linked into centrosymmetric dimers by pairs of O—H⋯O hydrogen bonds between one of two coordinating water mol­ecules (O5*W*) and the acetate O1 atom in an 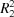
(8) manner. The second coordinating water mol­ecule (O6*W*) acts as hydrogen-atom donor, forming hydrogen bonds with the non-coordinating water mol­ecule and the nitrate anion, as shown in Fig. 1 ▸. The acetate O atoms act as acceptors in the hydrogen bonds with the H*N* groups of adjacent complex cation. Furthermore, the non-coordinating water mol­ecule forms hydrogen bonds to the nitrate anions. There are also some C—H⋯O contacts, which contribute to the crystal architecture and may be considered as weak hydrogen bonds (Fig. 2 ▸, Table 2 ▸).

## Database survey   

The ligand 1-(pyridin-2-yl­methyl­idene)-2-(pyridin-2-yl)hydrazine has been widely used in coordination chemistry. The current release of the CSD (Version 5.38, November 2016 + 1 update; Groom *et al.*, 2016 ▸) gave 16 hits. Six examples of complexes of the above ligand with *f*-block metal ions are known from the literature (Baraniak *et al.*, 1976 ▸; Ndiaye-Gueye, Dieng, Thiam, Sow *et al.*, 2017 ▸; Ndiaye-Gueye, Dieng, Thiam, Lo *et al.*, 2017 ▸; Ndiaye-Gueye, Dieng, Lo *et al.*, 2017 ▸). The other entries are related to complexes with *p*- and *d*-block metal ions. Structures are available for Ca^2+^ (Vantomme *et al.*, 2014 ▸), Cu^2+^ (Mesa *et al.*, 1988 ▸, 1989 ▸; Rojo *et al.*, 1988 ▸; Ainscough *et al.*, 1996 ▸; Chowdhury *et al.*, 2009 ▸; Mukherjee *et al.*, 2010 ▸; Chang *et al.*, 2011 ▸), Co^2+^ (Gerloch *et al.*, 1966 ▸), Ni^2+^ (Chiumia *et al.*, 1999 ▸) and Zn^2+^ (Dumitru *et al.*, 2005 ▸). In 15 cases, the ligand acts in a tridentate mode through the soft nitro­gen atoms of two pyridine rings and the imino function. The hard protonated nitro­gen atom remains non-coordinating in all known complexes.

## Synthesis and crystallization   

A mixture of 2-hydrazino­pyridine (1 mmol) and 2-pyridine­carbaldehyde (1 mmol) in ethanol (15 mL) was stirred under reflux during 30 min. A mixture of sodium acetate (3 mmol) and Tb(NO_3_)_3_·6H_2_O (1 mmol) in ethanol (10 mL) was added to the solution. The mixture was stirred for 30 min and the resulting yellow solution was filtered and the filtrate was kept at 298 K. A yellow powder appeared after one day and was collected by filtration. [C_15_H_20_TbN_4_O_6_]NO_3_·H_2_O. Yield 62%. Analysis calculated C, 30.47; H, 3.75; N, 11.84. Found: C, 30.42; H, 3.69; N, 11.89%. μ_eff_ (μB): 2.51. Λ_M_ (*S* cm^2^ mol^−1^): 90. IR (cm^−1^): 3225, 1588, 1575, 1558, 1445,1365, 820. δ_H_ (250 MHz, DMSO-*d*_6_) 11.21 (H, *s*, H-N-N); 8.54 (1H, *s*, H-Py); 8.16 (1H, H-Py); 8.10 (1H, *s*, H—C=N); 8.01 (1H, *d*, *J* = 7.50 Hz, H-Py); 7.81 (1H, *d*, *J* = 8 Hz, H-Py); 7.69 (1H, *d*, *J* = 8Hz, H-Py); 7.35 (2H, *d*, *J* = 8 Hz, H-Py); 6.83 (1H, *s*, H-Py); 4.722 (*s*, broad, H_2_O). δ_C_ (250 MHz, DMSO-*d*_6_): 106.526 (C-8), 115.559 (C-10), 118.845 (C-4), 122.912 (C-2), 136.465 (C-3), 138.015 (C-9), 139.171 (C-6), 147.804 (C-11).

## Refinement   

Crystal data, data collection and structure refinement details are summarized in Table 3 ▸. H atoms of the water mol­ecules were located in difference-Fourier maps. The O—H distances involving the O5*W* and O7*W* water mol­ecules were restrained to 0.82 (2) Å, those involving O6*W* were constrained using the AFIX 7 instruction. Other H atoms (CH and CH_3_ groups) were positioned geometrically and refined using a riding model with *U*_iso_(H) = 1.2*U*_eq_(C) (1.5 for CH_3_ groups). The chain bridging the two pyridine rings was found to be disordered. This disorder may be explained by the fact that the sequence of atoms C(py)—CH=N—NH—C(py) overlaps with the sequence C(py)—NH—N=CH—C(py), meaning two orientations for the ligand. For the refinement, we assumed that the C atom of CH group from one chain is situated nearby the N atom of NH group from the second chain, and the same relates inversely, whereas the imino N atoms of both chains occupy the same position. The occupancy factors were refined to a 0.52 (3):0.48 (3) ratio.

## Supplementary Material

Crystal structure: contains datablock(s) I. DOI: 10.1107/S2056989017009653/yk2107sup1.cif

Structure factors: contains datablock(s) I. DOI: 10.1107/S2056989017009653/yk2107Isup2.hkl

CCDC reference: 1511899

Additional supporting information:  crystallographic information; 3D view; checkCIF report

## Figures and Tables

**Table 1:** Selected bond lengths (Å)

Tb1—O5*W*	2.357 (3)	N2—C6	1.293 (17)
Tb1—O6*W*	2.362 (3)	N2—C6*A*	1.319 (13)
Tb1—O2	2.401 (3)	N2—N4	1.393 (13)
Tb1—O4	2.447 (3)	N2—N4*A*	1.396 (13)
Tb1—O3	2.476 (3)	N4—C5	1.417 (12)
Tb1—O1	2.476 (3)	C5—C6*A*	1.484 (14)
Tb1—N2	2.542 (4)	C6—C7	1.513 (17)
Tb1—N3	2.574 (4)	C7—N4*A*	1.410 (13)
Tb1—N1	2.588 (4)		

**Table 2:** Hydrogen-bond geometry (Å, °)

*D*—H⋯*A*	*D*—H	H⋯*A*	*D*⋯*A*	*D*—H⋯*A*
O5*W*—H5*WA*⋯O1^i^	0.82 (2)	1.91 (2)	2.718 (4)	175 (6)
O5*W*—H5*WB*⋯O9^ii^	0.80 (2)	2.12 (2)	2.849 (5)	153 (5)
O5*W*—H5*WB*⋯O3	0.80 (2)	2.48 (2)	2.874 (5)	112 (4)
O6*W*—H6*WA*⋯O7*W*	0.86	1.84	2.653 (5)	157
O6*W*—H6*WB*⋯O8	0.86	2.03	2.778 (5)	145
O7*W*—H7*WA*⋯O10^iii^	0.82 (2)	2.16 (2)	2.975 (6)	172 (6)
O7*W*—H7*WB*⋯O9^ii^	0.81 (2)	2.13 (2)	2.924 (6)	164 (8)
N4—H4*N*⋯O4^iv^	0.86	2.11	2.938 (11)	163
N4*A*—H4*NA*⋯O2^v^	0.86	2.04	2.896 (14)	179
C2—H2⋯O10^i^	0.93	2.58	3.407 (7)	148
C11—H11⋯O6*W*	0.93	2.52	3.125 (7)	123
C13—H13*C*⋯O8^ii^	0.96	2.47	3.227 (7)	136

Symmetry codes: (i) 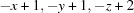; (ii) 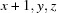; (iii) 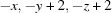; (iv) 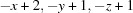; (v) 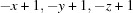.

**Table 3:** Experimental details

Crystal data	
Chemical formula	[Tb(C_2_H_3_O_2_)_2_(C_11_H_10_N_4_)(H_2_O)_2_]NO_3_·H_2_O
*M*_r_	591.29
Crystal system, space group	Triclinic, *P* 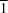
Temperature (K)	293
*a*, *b*, *c* (Å)	7.9184 (7), 11.7686 (10), 12.5196 (10)
α, β, γ (°)	78.981 (7), 73.965 (7), 72.222 (8)
*V* (Å^3^)	1060.45 (16)
*Z*	2
Radiation type	Mo *K*α
μ (mm^−1^)	3.40
Crystal size (mm)	0.08 × 0.07 × 0.05

Data collection	
Diffractometer	Bruker Kappa APEXII CCD
Absorption correction	Multi-scan (*SADABS*; Bruker, 2016 ▸)
*T*_min_, *T*_max_	0.230, 1.000
No. of measured, independent and observed [*I* > 2σ(*I*)] reflections	15646, 5172, 4355
*R*_int_	0.069
(sin θ/λ)_max_ (Å^−1^)	0.706

Refinement	
*R*[*F* ^2^ > 2σ(*F* ^2^)], *wR*(*F* ^2^), *S*	0.039, 0.083, 0.97
No. of reflections	5172
No. of parameters	314
No. of restraints	7
H-atom treatment	H atoms treated by a mixture of independent and constrained refinement
Δρ_max_, Δρ_min_ (e Å^−3^)	1.12, −1.58

Computer programs: *APEX3* and *SAINT* (Bruker, 2016 ▸), *SHELXT* (Sheldrick, 2015*a* ▸), *SHELXL2014* (Sheldrick, 2015*b* ▸) and *ORTEP-3 for Windows* (Farrugia, 2012 ▸).

## supplementary crystallographic information

### Crystal data

**Table:**

[Tb(C_2_H_3_O_2_)_2_(C_11_H_10_N_4_)(H_2_O)_2_]NO_3_·H_2_O	*Z* = 2
*M**_r_* = 591.29	*F*(000) = 584
Triclinic, *P*1	*D*_x_ = 1.852 Mg m^−^^3^
*a* = 7.9184 (7) Å	Mo *K*α radiation, λ = 0.71073 Å
*b* = 11.7686 (10) Å	Cell parameters from 9920 reflections
*c* = 12.5196 (10) Å	θ = 2.4–28.6°
α = 78.981 (7)°	µ = 3.40 mm^−^^1^
β = 73.965 (7)°	*T* = 293 K
γ = 72.222 (8)°	Prismatic, yellow
*V* = 1060.45 (16) Å^3^	0.08 × 0.07 × 0.05 mm

### Data collection

**Table:**

Bruker Kappa APEXII CCD diffractometer	5172 independent reflections
Radiation source: fine-focus sealed tube	4355 reflections with *I* > 2σ(*I*)
Detector resolution: 9 pixels mm^-1^	*R*_int_ = 0.069
CCD scans	θ_max_ = 30.1°, θ_min_ = 4.2°
Absorption correction: multi-scan (SADABS; Bruker, 2016)	*h* = −10→11
*T*_min_ = 0.230, *T*_max_ = 1.000	*k* = −16→14
15646 measured reflections	*l* = −17→16

### Refinement

**Table:**

Refinement on *F*^2^	Primary atom site location: structure-invariant direct methods
Least-squares matrix: full	Secondary atom site location: difference Fourier map
*R*[*F*^2^ > 2σ(*F*^2^)] = 0.039	Hydrogen site location: inferred from neighbouring sites
*wR*(*F*^2^) = 0.083	H atoms treated by a mixture of independent and constrained refinement
*S* = 0.97	*w* = 1/[σ^2^(*F*_o_^2^) + (0.0354*P*)^2^] where *P* = (*F*_o_^2^ + 2*F*_c_^2^)/3
5172 reflections	(Δ/σ)_max_ = 0.002
314 parameters	Δρ_max_ = 1.12 e Å^−^^3^
7 restraints	Δρ_min_ = −1.58 e Å^−^^3^

### Special details

**Table:**

Geometry. All esds (except the esd in the dihedral angle between two l.s. planes) are estimated using the full covariance matrix. The cell esds are taken into account individually in the estimation of esds in distances, angles and torsion angles; correlations between esds in cell parameters are only used when they are defined by crystal symmetry. An approximate (isotropic) treatment of cell esds is used for estimating esds involving l.s. planes.

### Fractional atomic coordinates and isotropic or equivalent isotropic displacement parameters (Å^2^)

**Table:**

	*x*	*y*	*z*	*U*_iso_*/*U*_eq_	Occ. (<1)
Tb1	0.57850 (2)	0.62098 (2)	0.74087 (2)	0.02775 (8)	
O1	0.3829 (4)	0.5115 (3)	0.8860 (2)	0.0386 (7)	
O2	0.3445 (4)	0.5452 (3)	0.7157 (3)	0.0425 (7)	
O3	0.6931 (4)	0.7991 (3)	0.7223 (3)	0.0440 (7)	
O4	0.8873 (4)	0.6407 (3)	0.6551 (3)	0.0497 (8)	
O5W	0.6728 (5)	0.6114 (3)	0.9059 (3)	0.0431 (7)	
H5WA	0.650 (8)	0.577 (4)	0.969 (2)	0.065*	
H5WB	0.705 (7)	0.670 (3)	0.903 (4)	0.065*	
O6W	0.3256 (4)	0.7749 (3)	0.8163 (3)	0.0473 (8)	
H6WA	0.359185	0.817289	0.851388	0.071*	
H6WB	0.243852	0.743930	0.862435	0.071*	
O7W	0.3776 (6)	0.9555 (4)	0.8901 (4)	0.0793 (14)	
H7WA	0.308 (7)	0.988 (7)	0.944 (4)	0.119*	
H7WB	0.480 (5)	0.925 (7)	0.901 (6)	0.119*	
O8	−0.0192 (5)	0.7371 (3)	0.8970 (4)	0.0629 (10)	
O9	−0.2955 (5)	0.8218 (4)	0.9709 (4)	0.0666 (11)	
O10	−0.0942 (6)	0.9208 (4)	0.9267 (4)	0.0735 (12)	
N1	0.7632 (5)	0.3976 (3)	0.7525 (3)	0.0368 (8)	
N2	0.7066 (6)	0.5259 (4)	0.5591 (3)	0.0547 (11)	
N3	0.4996 (5)	0.7501 (3)	0.5616 (3)	0.0362 (8)	
N4	0.858 (3)	0.4265 (11)	0.5572 (9)	0.043 (4)	0.48 (3)
H4N	0.946967	0.413835	0.499405	0.051*	0.48 (3)
N5	−0.1358 (5)	0.8281 (4)	0.9303 (4)	0.0440 (9)	
C1	0.7881 (6)	0.3285 (4)	0.8487 (4)	0.0437 (10)	
H1	0.726961	0.361070	0.915405	0.052*	
C2	0.8978 (8)	0.2133 (5)	0.8545 (5)	0.0564 (13)	
H2	0.906526	0.168655	0.923565	0.068*	
C3	0.9929 (10)	0.1656 (6)	0.7589 (6)	0.081 (2)	
H3	1.071840	0.088663	0.760515	0.097*	
C4	0.9705 (12)	0.2330 (6)	0.6591 (6)	0.112 (3)	
H4	1.034318	0.201991	0.591967	0.134*	
C5	0.8520 (8)	0.3480 (5)	0.6585 (4)	0.0620 (15)	
C6	0.712 (3)	0.5933 (16)	0.4644 (13)	0.041 (3)	0.48 (3)
H6	0.804961	0.573789	0.401055	0.049*	0.48 (3)
C7	0.5569 (8)	0.7066 (5)	0.4646 (4)	0.0549 (13)	
C8	0.5096 (12)	0.7717 (7)	0.3673 (5)	0.095 (3)	
H8	0.548346	0.736391	0.301208	0.114*	
C9	0.4057 (9)	0.8881 (6)	0.3695 (6)	0.0730 (18)	
H9	0.375238	0.933981	0.304892	0.088*	
C10	0.3486 (9)	0.9347 (5)	0.4675 (6)	0.0726 (17)	
H10	0.276887	1.013352	0.472204	0.087*	
C11	0.3980 (9)	0.8641 (5)	0.5604 (5)	0.0653 (16)	
H11	0.357923	0.897893	0.627291	0.078*	
C12	0.8521 (5)	0.7497 (4)	0.6718 (4)	0.0337 (9)	
C13	0.9806 (7)	0.8089 (5)	0.6350 (5)	0.0532 (13)	
H13A	0.925944	0.891305	0.611002	0.080*	
H13B	1.070322	0.772725	0.573214	0.080*	
H13C	1.038015	0.805322	0.694267	0.080*	
C14	0.2996 (6)	0.5032 (4)	0.8165 (4)	0.0358 (9)	
C15	0.1467 (7)	0.4436 (5)	0.8522 (5)	0.0532 (13)	
H15A	0.053392	0.483710	0.910406	0.080*	
H15B	0.192431	0.360901	0.879482	0.080*	
H15C	0.096670	0.448175	0.789338	0.080*	
C6A	0.782 (3)	0.4094 (12)	0.5581 (11)	0.039 (3)	0.52 (3)
H6A	0.790310	0.368827	0.499134	0.046*	0.52 (3)
N4A	0.638 (3)	0.5818 (11)	0.4654 (10)	0.043 (4)	0.52 (3)
H4NA	0.643634	0.543027	0.412190	0.052*	0.52 (3)

### Atomic displacement parameters (Å^2^)

**Table:**

	*U*^11^	*U*^22^	*U*^33^	*U*^12^	*U*^13^	*U*^23^
Tb1	0.02887 (12)	0.03134 (13)	0.02209 (11)	−0.00834 (8)	−0.00363 (8)	−0.00384 (8)
O1	0.0436 (16)	0.0513 (19)	0.0251 (15)	−0.0198 (14)	−0.0081 (13)	−0.0027 (14)
O2	0.0512 (18)	0.057 (2)	0.0283 (16)	−0.0294 (16)	−0.0129 (14)	0.0028 (15)
O3	0.0420 (17)	0.0430 (18)	0.046 (2)	−0.0133 (14)	−0.0061 (15)	−0.0061 (15)
O4	0.0369 (17)	0.052 (2)	0.051 (2)	−0.0125 (15)	0.0051 (15)	−0.0068 (17)
O5W	0.061 (2)	0.051 (2)	0.0276 (16)	−0.0307 (16)	−0.0136 (15)	0.0012 (14)
O6W	0.0379 (16)	0.0488 (19)	0.052 (2)	−0.0117 (14)	0.0042 (15)	−0.0190 (17)
O7W	0.063 (3)	0.073 (3)	0.102 (4)	0.002 (2)	−0.016 (3)	−0.049 (3)
O8	0.055 (2)	0.049 (2)	0.075 (3)	−0.0056 (18)	0.0004 (19)	−0.020 (2)
O9	0.042 (2)	0.073 (3)	0.082 (3)	−0.0155 (19)	−0.0052 (19)	−0.016 (2)
O10	0.084 (3)	0.051 (2)	0.093 (3)	−0.029 (2)	−0.017 (2)	−0.012 (2)
N1	0.0383 (19)	0.038 (2)	0.032 (2)	−0.0072 (16)	−0.0093 (15)	−0.0028 (16)
N2	0.077 (3)	0.042 (2)	0.025 (2)	0.009 (2)	−0.008 (2)	−0.0065 (18)
N3	0.0401 (19)	0.0336 (19)	0.033 (2)	−0.0116 (16)	−0.0075 (16)	0.0009 (16)
N4	0.049 (8)	0.037 (6)	0.028 (5)	−0.005 (5)	0.009 (5)	−0.005 (4)
N5	0.046 (2)	0.042 (2)	0.046 (2)	−0.0120 (19)	−0.0131 (18)	−0.0086 (19)
C1	0.053 (3)	0.042 (3)	0.036 (3)	−0.016 (2)	−0.010 (2)	0.003 (2)
C2	0.071 (3)	0.046 (3)	0.052 (3)	−0.014 (3)	−0.027 (3)	0.011 (3)
C3	0.097 (5)	0.049 (3)	0.067 (4)	0.024 (3)	−0.023 (4)	−0.002 (3)
C4	0.152 (7)	0.065 (4)	0.055 (4)	0.056 (5)	−0.011 (4)	−0.015 (3)
C5	0.082 (4)	0.047 (3)	0.032 (3)	0.014 (3)	−0.007 (3)	−0.007 (2)
C6	0.045 (8)	0.046 (7)	0.026 (6)	−0.002 (7)	−0.005 (6)	−0.010 (5)
C7	0.074 (4)	0.050 (3)	0.032 (3)	−0.005 (3)	−0.017 (2)	0.003 (2)
C8	0.153 (7)	0.074 (4)	0.036 (3)	0.014 (5)	−0.040 (4)	−0.003 (3)
C9	0.094 (5)	0.064 (4)	0.058 (4)	−0.011 (3)	−0.041 (4)	0.016 (3)
C10	0.089 (4)	0.046 (3)	0.067 (4)	−0.001 (3)	−0.024 (3)	0.013 (3)
C11	0.085 (4)	0.044 (3)	0.048 (3)	0.004 (3)	−0.011 (3)	−0.002 (3)
C12	0.031 (2)	0.044 (2)	0.024 (2)	−0.0123 (18)	−0.0048 (17)	0.0010 (18)
C13	0.055 (3)	0.055 (3)	0.045 (3)	−0.027 (3)	0.001 (2)	0.006 (2)
C14	0.039 (2)	0.039 (2)	0.030 (2)	−0.0115 (19)	−0.0073 (18)	−0.0023 (19)
C15	0.049 (3)	0.069 (4)	0.049 (3)	−0.031 (3)	−0.013 (2)	0.002 (3)
C6A	0.046 (7)	0.037 (6)	0.032 (5)	−0.012 (5)	−0.001 (5)	−0.013 (4)
N4A	0.067 (10)	0.039 (5)	0.029 (5)	−0.009 (6)	−0.026 (6)	−0.006 (4)

### Geometric parameters (Å, º)

**Table:**

Tb1—O5W	2.357 (3)	N4—C5	1.417 (12)
Tb1—O6W	2.362 (3)	N4—H4N	0.8600
Tb1—O2	2.401 (3)	C1—C2	1.370 (7)
Tb1—O4	2.447 (3)	C1—H1	0.9300
Tb1—O3	2.476 (3)	C2—C3	1.349 (9)
Tb1—O1	2.476 (3)	C2—H2	0.9300
Tb1—N2	2.542 (4)	C3—C4	1.371 (10)
Tb1—N3	2.574 (4)	C3—H3	0.9300
Tb1—N1	2.588 (4)	C4—C5	1.392 (8)
Tb1—C14	2.812 (4)	C4—H4	0.9300
Tb1—C12	2.865 (4)	C5—C6A	1.484 (14)
Tb1—Tb1^i^	6.5113 (7)	C6—C7	1.513 (17)
O1—C14	1.261 (5)	C6—H6	0.9300
O2—C14	1.259 (5)	C7—C8	1.390 (8)
O3—C12	1.256 (5)	C7—N4A	1.410 (13)
O4—C12	1.272 (6)	C8—C9	1.367 (9)
O5W—H5WA	0.819 (19)	C8—H8	0.9300
O5W—H5WB	0.800 (19)	C9—C10	1.345 (9)
O6W—H6WA	0.8617	C9—H9	0.9300
O6W—H6WB	0.8617	C10—C11	1.370 (8)
O7W—H7WA	0.82 (2)	C10—H10	0.9300
O7W—H7WB	0.81 (2)	C11—H11	0.9300
O8—N5	1.236 (5)	C12—C13	1.336 (6)
O9—N5	1.245 (5)	C13—H13A	0.9600
O10—N5	1.223 (5)	C13—H13B	0.9600
N1—C5	1.332 (6)	C13—H13C	0.9600
N1—C1	1.345 (6)	C14—C15	1.502 (6)
N2—C6	1.293 (17)	C15—H15A	0.9600
N2—C6A	1.319 (13)	C15—H15B	0.9600
N2—N4	1.393 (13)	C15—H15C	0.9600
N2—N4A	1.396 (13)	C6A—H6A	0.9300
N3—C7	1.317 (6)	N4A—H4NA	0.8600
N3—C11	1.337 (6)		
			
O5W—Tb1—O6W	85.06 (12)	C6A—N2—N4A	114.0 (9)
O5W—Tb1—O2	128.75 (11)	C6—N2—Tb1	119.8 (8)
O6W—Tb1—O2	82.50 (11)	C6A—N2—Tb1	121.7 (6)
O5W—Tb1—O4	81.62 (12)	N4—N2—Tb1	116.2 (6)
O6W—Tb1—O4	125.77 (11)	N4A—N2—Tb1	119.5 (6)
O2—Tb1—O4	142.99 (12)	C7—N3—C11	115.7 (4)
O5W—Tb1—O3	72.94 (11)	C7—N3—Tb1	121.4 (3)
O6W—Tb1—O3	73.67 (11)	C11—N3—Tb1	122.9 (3)
O2—Tb1—O3	146.48 (11)	N2—N4—C5	114.3 (9)
O4—Tb1—O3	52.15 (11)	N2—N4—H4N	122.8
O5W—Tb1—O1	75.65 (10)	C5—N4—H4N	122.8
O6W—Tb1—O1	76.13 (11)	O10—N5—O8	120.8 (4)
O2—Tb1—O1	53.11 (10)	O10—N5—O9	120.8 (4)
O4—Tb1—O1	146.94 (11)	O8—N5—O9	118.4 (4)
O3—Tb1—O1	137.72 (10)	N1—C1—C2	124.1 (5)
O5W—Tb1—N2	137.14 (13)	N1—C1—H1	117.9
O6W—Tb1—N2	137.52 (13)	C2—C1—H1	117.9
O2—Tb1—N2	73.60 (13)	C3—C2—C1	119.1 (5)
O4—Tb1—N2	69.39 (14)	C3—C2—H2	120.5
O3—Tb1—N2	109.07 (14)	C1—C2—H2	120.5
O1—Tb1—N2	113.20 (13)	C2—C3—C4	118.5 (5)
O5W—Tb1—N3	146.84 (11)	C2—C3—H3	120.8
O6W—Tb1—N3	78.75 (12)	C4—C3—H3	120.8
O2—Tb1—N3	77.73 (11)	C3—C4—C5	119.8 (6)
O4—Tb1—N3	84.69 (12)	C3—C4—H4	120.1
O3—Tb1—N3	74.79 (11)	C5—C4—H4	120.1
O1—Tb1—N3	126.77 (10)	N1—C5—C4	122.0 (5)
N2—Tb1—N3	62.34 (12)	N1—C5—N4	116.7 (6)
O5W—Tb1—N1	81.87 (12)	C4—C5—N4	119.5 (6)
O6W—Tb1—N1	149.42 (12)	N1—C5—C6A	115.0 (6)
O2—Tb1—N1	84.37 (11)	C4—C5—C6A	121.3 (7)
O4—Tb1—N1	79.43 (11)	N2—C6—C7	114.8 (11)
O3—Tb1—N1	127.41 (10)	N2—C6—H6	122.6
O1—Tb1—N1	73.90 (11)	C7—C6—H6	122.6
N2—Tb1—N1	62.72 (12)	N3—C7—C8	123.0 (5)
N3—Tb1—N1	124.96 (11)	N3—C7—N4A	117.3 (6)
O5W—Tb1—C14	102.28 (12)	C8—C7—N4A	118.4 (7)
O6W—Tb1—C14	77.74 (12)	N3—C7—C6	112.5 (8)
O2—Tb1—C14	26.47 (11)	C8—C7—C6	122.9 (8)
O4—Tb1—C14	156.49 (12)	C9—C8—C7	119.5 (6)
O3—Tb1—C14	151.29 (12)	C9—C8—H8	120.3
O1—Tb1—C14	26.64 (11)	C7—C8—H8	120.3
N2—Tb1—C14	93.73 (14)	C10—C9—C8	118.3 (6)
N3—Tb1—C14	102.27 (12)	C10—C9—H9	120.9
N1—Tb1—C14	78.22 (12)	C8—C9—H9	120.9
O5W—Tb1—C12	76.23 (12)	C9—C10—C11	118.8 (6)
O6W—Tb1—C12	99.55 (12)	C9—C10—H10	120.6
O2—Tb1—C12	154.91 (11)	C11—C10—H10	120.6
O4—Tb1—C12	26.23 (12)	N3—C11—C10	124.7 (6)
O3—Tb1—C12	25.92 (11)	N3—C11—H11	117.6
O1—Tb1—C12	151.81 (11)	C10—C11—H11	117.6
N2—Tb1—C12	89.05 (14)	O3—C12—O4	117.7 (4)
N3—Tb1—C12	78.17 (11)	O3—C12—C13	121.8 (5)
N1—Tb1—C12	103.96 (12)	O4—C12—C13	120.5 (4)
C14—Tb1—C12	177.06 (12)	O3—C12—Tb1	59.5 (2)
O5W—Tb1—Tb1^i^	41.12 (8)	O4—C12—Tb1	58.2 (2)
O6W—Tb1—Tb1^i^	81.29 (9)	C13—C12—Tb1	177.5 (4)
O2—Tb1—Tb1^i^	87.80 (7)	C12—C13—H13A	109.5
O4—Tb1—Tb1^i^	117.10 (9)	C12—C13—H13B	109.5
O3—Tb1—Tb1^i^	110.91 (8)	H13A—C13—H13B	109.5
O1—Tb1—Tb1^i^	34.82 (6)	C12—C13—H13C	109.5
N2—Tb1—Tb1^i^	130.93 (10)	H13A—C13—H13C	109.5
N3—Tb1—Tb1^i^	156.65 (8)	H13B—C13—H13C	109.5
N1—Tb1—Tb1^i^	70.70 (8)	O2—C14—O1	119.8 (4)
C14—Tb1—Tb1^i^	61.39 (9)	O2—C14—C15	119.2 (4)
C12—Tb1—Tb1^i^	117.28 (8)	O1—C14—C15	120.9 (4)
C14—O1—Tb1	91.7 (3)	O2—C14—Tb1	58.2 (2)
C14—O2—Tb1	95.3 (2)	O1—C14—Tb1	61.7 (2)
C12—O3—Tb1	94.6 (3)	C15—C14—Tb1	177.2 (3)
C12—O4—Tb1	95.5 (2)	C14—C15—H15A	109.5
Tb1—O5W—H5WA	134 (4)	C14—C15—H15B	109.5
Tb1—O5W—H5WB	109 (4)	H15A—C15—H15B	109.5
H5WA—O5W—H5WB	113 (3)	C14—C15—H15C	109.5
Tb1—O6W—H6WA	110.0	H15A—C15—H15C	109.5
Tb1—O6W—H6WB	109.9	H15B—C15—H15C	109.5
H6WA—O6W—H6WB	108.8	N2—C6A—C5	114.7 (9)
H7WA—O7W—H7WB	112 (4)	N2—C6A—H6A	122.7
C5—N1—C1	116.4 (4)	C5—C6A—H6A	122.7
C5—N1—Tb1	119.4 (3)	N2—N4A—C7	115.0 (8)
C1—N1—Tb1	124.0 (3)	N2—N4A—H4NA	122.5
C6—N2—N4	112.9 (10)	C7—N4A—H4NA	122.5

Symmetry code: (i) −*x*+1, −*y*+1, −*z*+2.

### Hydrogen-bond geometry (Å, º)

**Table:**

*D*—H···*A*	*D*—H	H···*A*	*D*···*A*	*D*—H···*A*
O5*W*—H5*WA*···O1^i^	0.82 (2)	1.91 (2)	2.718 (4)	175 (6)
O5*W*—H5*WB*···O9^ii^	0.80 (2)	2.12 (2)	2.849 (5)	153 (5)
O5*W*—H5*WB*···O3	0.80 (2)	2.48 (2)	2.874 (5)	112 (4)
O6*W*—H6*WA*···O7*W*	0.86	1.84	2.653 (5)	157
O6*W*—H6*WB*···O8	0.86	2.03	2.778 (5)	145
O7*W*—H7*WA*···O10^iii^	0.82 (2)	2.16 (2)	2.975 (6)	172 (6)
O7*W*—H7*WB*···O9^ii^	0.81 (2)	2.13 (2)	2.924 (6)	164 (8)
N4—H4*N*···O4^iv^	0.86	2.11	2.938 (11)	163
N4*A*—H4*NA*···O2^v^	0.86	2.04	2.896 (14)	179
C2—H2···O10^i^	0.93	2.58	3.407 (7)	148
C11—H11···O6*W*	0.93	2.52	3.125 (7)	123
C13—H13*C*···O8^ii^	0.96	2.47	3.227 (7)	136

Symmetry codes: (i) −*x*+1, −*y*+1, −*z*+2; (ii) *x*+1, *y*, *z*; (iii) −*x*, −*y*+2, −*z*+2; (iv) −*x*+2, −*y*+1, −*z*+1; (v) −*x*+1, −*y*+1, −*z*+1.
